# Optimization of HIV testing services in Germany using HIV indicator diseases: study protocol of the HeLP study

**DOI:** 10.1186/s13690-023-01161-9

**Published:** 2023-08-25

**Authors:** Frederik Valbert, Uwe Koppe, Daniel Schmidt, Amrei Krings, Barbara Gunsenheimer-Bartmeyer, Patrik Dröge, Thomas Ruhnke, Georg Behrens, Markus Bickel, Christoph Boesecke, Stefan Esser, Jürgen Wasem, Anja Neumann

**Affiliations:** 1 Institute for Healthcare Management and Research, University of Duisburg-Essen, Essen, Germany; 2 Department of Infectious Disease Epidemiology, Robert Koch Institute, Berlin, Germany; 3 AOK Research Institute (WIdO), Berlin, Germany; 4 Department for Rheumatology and Immunology, Hannover Medical School, Hanover, Germany; 5 German Centre for Infection Research (DZIF), Bonn, Germany; 6 Infektiologikum Frankfurt, Frankfurt, Germany; 7 Department of Medicine I, Bonn University Hospital, Bonn, Germany; 8 Department of Dermatology and Venerology, University Hospital Essen, University Duisburg- Essen, Essen, Germany

**Keywords:** HIV, Human immunodeficiency virus, AIDS, Acquired immune deficiency syndrome, Indicator diseases, Testing, Diagnostic, Routine data, Mixed methods, Germany

## Abstract

**Background:**

Despite the potentially accompanying negative clinical, epidemiologic, and health economic effects, a large proportion of persons living with the human immunodeficiency virus (HIV) are diagnosed late. Internationally, numerous diseases are known to be HIV indicator diseases. Adequate HIV testing in the presence of HIV indicator diseases could help to diagnose unknown HIV infections earlier. The objective of the HeLP study is to validate published HIV indicator diseases for the German setting and to identify guidelines in terms of these indicator diseases in order to reduce knowledge gaps and increase HIV testing when HIV indicator diseases are diagnosed.

**Methods:**

A mixed methods approach is used. In a first step, published HIV indicator diseases will be identified in a systematic literature review and subsequently discussed with clinical experts regarding their relevance for the German setting. For the validation of selected indicator diseases different data sets (two cohort studies, namely HIV-1 seroconverter study & ClinSurv-HIV, and statutory health insurance routine data) will be analyzed. Sensitivity analyses using different time periods will be performed. Guidelines of HIV indicator diseases validated in the HeLP study will be reviewed for mentioning HIV and for HIV testing recommendations. In addition, semi-standardized interviews (followed by a free discussion) with guideline creators will identify reasons why HIV testing recommendations were (not) included. Subsequently, a random sample of physicians in medical practices will be surveyed to identify how familiar physicians are with HIV testing recommendations in guidelines and, if so, which barriers are seen to perform the recommended tests in everyday care.

**Discussion:**

The HeLP-study adopts the challenge to validate published HIV indicator diseases for the German setting and has the potential to close a knowledge gap regarding this objective. This has the potential to improve targeted HIV testing for patients with HIV indicator diseases and consequently lead to earlier HIV diagnosis.

**Trial registration:**

DRKS00028743

## Background

According to the United Nations 95-95-95 targets, Germany aims to achieve that by 2025 more than 95% of people living with the human immunodeficiency virus (HIV) know their diagnosis [1]. So far, Germany has not achieved this target [2]. Delayed diagnosis can lead to negative effects from the clinical, epidemiological, and health economic perspectives [3–7]. Late HIV diagnosis is associated with higher morbidity and mortality on the individual patient level [3, 4]. Moreover, there is a risk of further virus transmission, which can be avoided with adequate therapy of the infection [5, 6]. In addition to the immediate negative consequences of a late diagnosis on the health and quality of life of individuals, the effects of HIV transmission in undiagnosed individuals also appear relevant from a health economic perspective. Since it is a chronic disease, lifelong therapy becomes necessary for every HIV infection. From a societal perspective, the costs of HIV in Germany are estimated around 20,000€ per patient and year [7]. In the case of an initial diagnosis in stage acquired immune deficiency syndrome (AIDS), these costs increase significantly [7].

Overall, it is estimated that 44-64% of HIV-positive people in Germany are diagnosed at an advanced stage of disease, hence they are considered late presenters according to the definition of Antinori et al. from 2011 (AIDS-defining disease and/or CD4 cell count below 350 per microliter at the time of initial diagnosis) (an updated consensus definition was published in November 2022) [7–13]. Among the estimated 2,400 people in Germany with an initial HIV diagnosis in 2021, the Robert Koch Institute (RKI) estimates that 790 people (33%) were diagnosed with advanced immunodeficiency (AIDS or CD4 cell count of less than 200) [2].

Adequate HIV testing in the presence of HIV indicator diseases has the potential to diagnose unknown infections earlier and thus be cost-effective [14]. HIV indicator diseases are diseases that occur particularly frequent in (undiagnosed) HIV-positive people. This is explained either by a similar transmission pathway or as a causal consequence of the HIV infection. While HIV indicator diseases have already been scientifically elaborated at the international level, there is still a lack of evidence specific to the German setting [15–18].

In the HIV testing recommendations in guidelines and practice study (German title of the study: “HIV-Testempfehlungen in Leitlinien und Praxis”; acronym: HeLP), published HIV indicator diseases from the international level will be validated for the German setting. Also, guidelines for the treatment or diagnosis of validated indicator diseases will be identified and analyzed with regard to the mention of HIV or the recommendation of HIV testing. In addition, reasons for (not) including HIV testing recommendations in the creation/updating of guidelines on HIV indicator diseases will be investigated. Furthermore, the awareness of such guidelines among practicing physicians as well as reasons for not carrying out the recommended HIV tests will be examined. Finally, measures to increase HIV testing rates for validated HIV indicator diseases will be explored.

## Methods

**Fig. 1:**
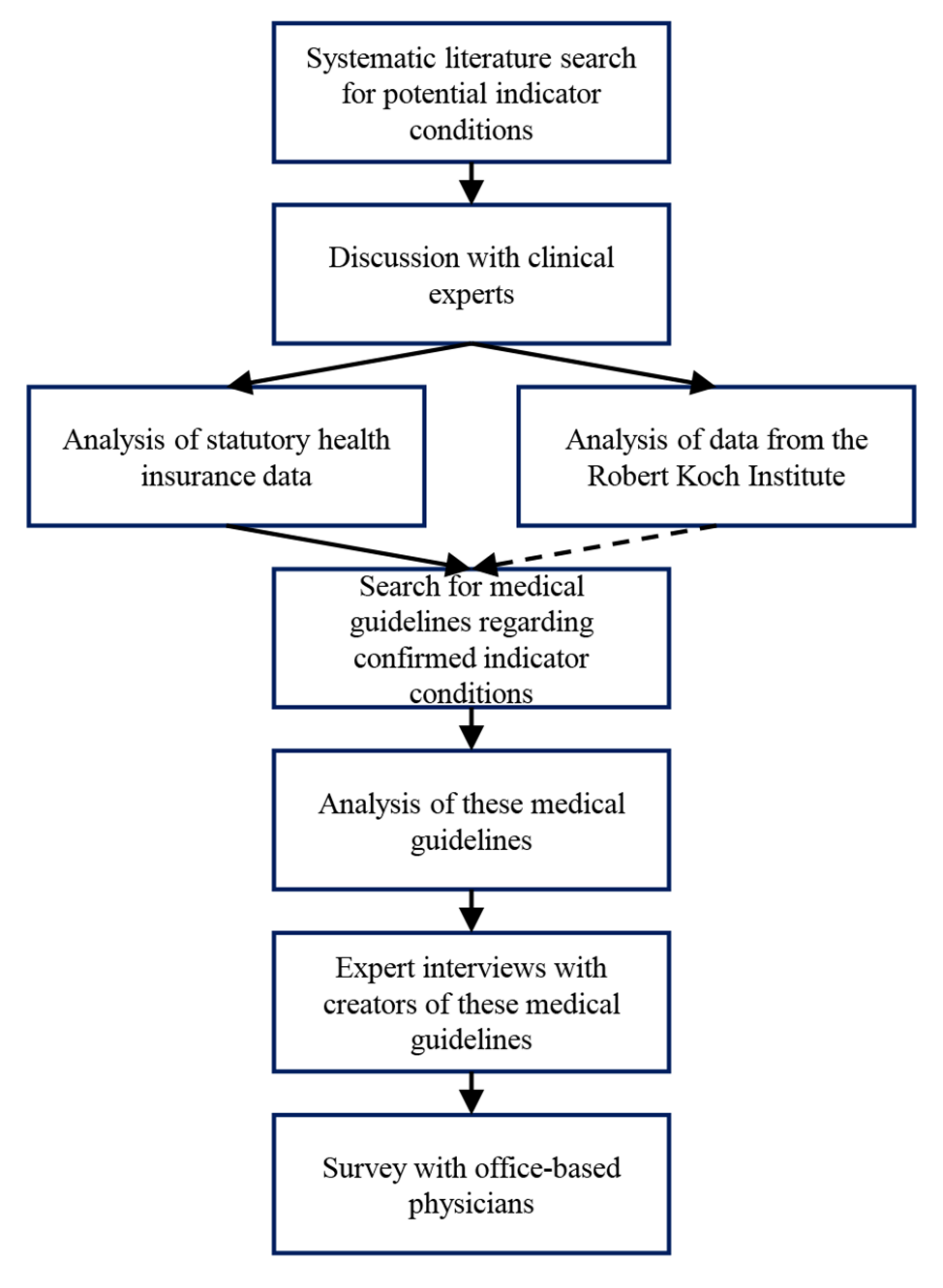
Simplified flow chart of the HeLP study

The HeLP study is publicly funded by the German Federal Joint Committee (G-BA, “Gemeinsamer Bundesausschuss”) as part of the Innovationsfonds program to further develop the German healthcare system based on the standards and principles of evidence-based healthcare (funding number 01VSF21050). A simplified overview of the study is shown in Fig. 1.

### Study design and research questions

Using a mixed methods approach (combination of quantitative and qualitative research methods), the HeLP study aims to address the following research questions resulting from the lack of evidence mentioned above: (Q_1a_) Can international HIV indicator diseases currently published in the literature be validated in routine data from statutory health insurances (SHI) and from HIV cohort data at the RKI (HIV-1 Seroconverter Study and ClinSurv-HIV)? (Q_1b_) What is the prevalence/incidence of HIV stratified by sex and age for indicator diseases in Germany? (Q_2_) Are guidelines available for the diagnosis or treatment of the indicator diseases validated in Q_1a_? (Q_3_) Is HIV mentioned in these guidelines? (Q_4_) Is HIV testing mentioned in these guidelines? (Q_5_) and furthermore, is it also recommended in these guidelines? (Q_6_) Why was the recommendation on HIV testing (not) included when guidelines were created/updated? (Q_7_) What potential hurdles do the authors and editors of guidelines including testing recommendations for validated HIV indicator diseases identify, if HIV testing is not always offered in practice? (Q_8_) Regarding guidelines on validated HIV indicator diseases with HIV testing recommendations, is the recommendation known by physicians in the outpatient-setting including general practitioners and specialists? (Q_9_) What reasons are seen by physicians in the outpatient-setting for not following known HIV testing recommendations? (Q_10_) What measures appear appropriate to increase HIV testing rates for the validated HIV indicator diseases?

### Identification of HIV indicator diseases

To identify potential HIV indicator diseases relevant in other western countries a systematic literature search will be done, using the medical bibliographic databases Medline and Embase. Titles and abstracts as well as full-texts of potentially relevant publications will be reviewed by two persons. Data will be extracted and a qualitative data synthesis of mentioned indicator diseases will be performed. Subsequently, the research results will be discussed with clinical experts to identify the most relevant indicator disease for the study (e.g., due to their epidemiological significance in Germany or their specificity with regard to HIV).

### Validation of HIV indicator diseases

To validate the HIV indicator diseases known in the literature, different data sets (HIV-1 seroconverter study, ClinSurv-HIV, SHI routine data) are analyzed. The SHI routine data will be transmitted for analysis in an anonymized form. Data of the HIV-1 seroconverter study and the ClinSurv-HIV study will be analyzed on site at the RKI and results are discussed in the project team only on an aggregated level. In the context of these analyses, HIV indicator diseases are classified as diseases that are particularly often accompanied by an HIV infection. This synchronous presence of HIV infections and HIV indicator diseases can be explained by similar transmission routes (e.g., sexually transmitted infections such as syphilis or gonorrhea) or as a causal sequence, in which the HIV indicator disease is a consequence of the weakened immune system caused by HIV (e.g., opportunistic infections such as candidiasis). Other possible reasons for classification as an indicator disease are: the disease is considered to be AIDS-defining in HIV-positive persons, or an undiagnosed HIV infection would have particularly negative effects on the health of the person affected by this disease. As part of the routine data analysis, the University of Duisburg-Essen in close cooperation with the consortium partners will analyze health insurance data focusing on the rate of HIV diagnoses (a) parallel to a present indicator disease and (b) in the years after the occurrence of the indicator disease. The relative proportion of HIV-positive people in the respective collectives of disease has to be investigated. Since an HIV infection is a chronic disease that can be treated but not cured, the HIV diagnosis in scenario (a) does not have to be present at the exact same time as the indicator disease, but HIV diagnoses coded in advance also prove that an HIV infection exists parallel with the indicator disease. The time periods during which an indicator disease is contextualized with HIV are determined based on published evidence/assumptions, because the exact timing of HIV transmission is not noted in routine data and is often unknown. Sensitivity analyses will be performed using different time periods. The analyses for detection of indicator diseases will also include stratification, for example by age group or gender. HIV testing is considered effective, if an (undiagnosed) HIV prevalence of at least 0.1% is observable in the respective (sub)groups (published studies differ on the classifications of conditions with a prevalence of exactly 0.1% and how strictly “undiagnosed” is considered) [14, 15]. Stratification is intended to test whether the HIV prevalence of at least 0.1% applies to the total population of people affected by an HIV indicator disease or whether the effect is observable only in individual subgroups. In order to perform the analyses in terms of the scenarios (a) and (b), the anonymized data set of the transmitted routine data includes, for example, data on demographic characteristics of the insured persons, data on diagnoses and, if applicable, treatment dates from the inpatient and outpatient sectors. To validate or falsify documented HIV diagnoses, data of prescribed drugs and German billing codes as well as diagnosis related groups can be used. The routine data (2016–2021) are supplied by the AOK Research Institute (WIdO), which were derived from statutory health insurance data of eleven regional AOK (“Allgemeine Ortskrankenkasse”) insurances covering about 27 million insured persons in total. At the RKI, the occurrence of indicator diseases is being analyzed in data from the two cohort studies, namely the HIV-1 Seroconverter Study as well as the ClinSurv-HIV study. The HIV-1 Seroconverter Study includes HIV positive persons with a known date of HIV infection who are prospectively followed [19–22]. The ClinSurv-HIV study includes HIV positive persons whose date of HIV infection is unknown [23, 24]. In total, 26,785 people are included in the two cohort studies. Because the studies have been started in 1997 and 1999, a total of 6,449 patients who were diagnosed with HIV but were followed for at least one year or longer without receiving antiretroviral therapy are included in the study. In the period with HIV infection but without antiretroviral therapy, the occurrence of diseases that would also occur in undetected HIV infections as a result of the course of HIV infection can be investigated, such as candidiasis or Kaposi sarcoma.

### Analysis of German medical guidelines regarding HIV indicator diseases

After the identification of validated indicator diseases for the German population, a structured literature search for German medical guidelines concerning their diagnosis or therapy will be conducted. For example, the portal of the Association of the Scientific Medical Societies in Germany (“Arbeitsgemeinschaft der Wissenschaftlichen Medizinischen Fachgesellschaften”, AWMF) as well as databases such as PubMed and Embase will be used. The guidelines will be screened for the mentioning of potential HIV infection as well as the recommendation for HIV testing. Both, this literature searches and the subsequent screening for HIV consideration will be conducted for quality assurance purposes using the four-eyes principle, in which each indicator disease and its guidelines will be examined by at least two partners of the consortium.

### Expert interviews on guideline composition

Semi-standardized interviews with guideline creators will be conducted by staff of the Institute for Healthcare Management and Research. Reasons for (not) recommending HIV testing in guidelines will be collected. The semi-standardized interviews are followed by a free discussion, which complements the semi-standardized interviews due to their exploratory nature. In addition to a description of the problem, approaches for solutions should be discussed as well. Following the approach of Krueger and Casey, the semi-structured expert interviews will be conducted by a team of moderators based on a semi-structured guideline, which will be prepared by the whole consortium in interdisciplinary cooperation [25]. The interviews will be recorded and transcribed afterwards. Then, a qualitative content analysis based on Mayring will be carried out using MAXQDA [26, 27].

### Survey with office-based physicians

A random sample of 1,960 physicians in medical practices will be contacted for a standardized and anonymous written survey. Participation will be possible by mail as well as online. This shall counteract the risk that the reduction to one participation option could cause a selection bias. In the course of a very conservatively case number planning, a response rate of 10% is assumed. Even though the analysis will focus on descriptive methods primarily, with the expected number of responses, it will be possible to examine characteristics of physicians and their willingness to perform an HIV test in a bivariate model. For example, if a bivariate model is used to search for the correlation of two characteristics in a subgroup with 25% of the study participants, a power of 95% is given for an effect size which is derived from a coefficient of determination of 0.2. To increase the motivation to participate, the use of incentives is planned. The specialty of the physicians contacted will depend on the results of the previous work packages. The objective of the written survey is to identify how familiar physicians are to HIV testing recommendations in guidelines of indicator diseases and, if so, are barriers seen to perform the recommended tests in everyday care. The questionnaire will be developed at the Institute for Healthcare Management and Research in close cooperation with all partners of the consortium. A pretest will be conducted. The results of the written survey will be analyzed using IBM SPSS Statistics at the Institute for Healthcare Management and Research. The interpretation of the results and their discussion will take place involving all consortium partners.

### Strategy derivation and dissemination

Based on the previous work packages, a list of validated indicator diseases for the German context will be published. Additionally, general approaches to increase the HIV testing rate in validated indicator diseases will be developed and scientifically published. Direct contacts to affected physicians is planned (e.g., via professional societies or professional press), so that an information campaign towards a clinically optimized and health-economically more effective HIV testing practice is carried out.

### Ethics and transparency

The HeLP study is financed by the Innovation Fund exclusively (Fund of the GBA, funding code: 01VSF21050). A positive ethics vote was obtained from the ethics committee of the Faculty of Medicine of the University of Duisburg-Essen (identifier: 22-10908-BO). The study was registered in the German Clinical Trials Register (identifier: DRKS00028743).

## Discussion

The HeLP-study adopts the challenge to validate published HIV indicator diseases and has the potential to close a lack of knowledge regarding this objective.

A potential risk that may endanger the achievement of the project’s objectives could be the possibility that no or only a few indicator diseases can be validated for Germany. This risk is countered by the very broad data base for the analyses. In addition, even in case of the unlikely result that indicator diseases cannot be validated for Germany, the HeLP-study would be associated with a valuable gain in knowledge.

A further risk can be if German medical guidelines even exist for the identified indicator diseases. However, preliminary research and interviews with experts confirm the existence of numerous relevant German guidelines, at least with regard to indicator diseases, which were identified at the European level by Raben et al. [16, 28].

Conservative planning of the number of cases in the survey with physicians minimizes the risk of missing the planned response rate. The use of incentives in the survey has an additional minimizing effect on this risk.

The HeLP study has the potential to improve medical care in Germany in several aspects.

A validation of the currently established HIV indicator diseases, including a stratification according to demographic characteristics, offers the chance to test for HIV infections in Germany in a more targeted way in the future. This more precise use of HIV testing may be suitable to increase the acceptance of HIV tests among physicians and patients. In addition, more precise testing offers advantages concerning early detection and health economic efficiency.

The analysis of the guidelines and the survey with physicians has the potential not only to describe hurdles in terms of HIV testing in care, but also, by publicizing these gaps and developing strategies for solutions, to achieve increased awareness and increase the HIV testing, when HIV indicator diseases are diagnosed.

The expert interviews can identify (probably generalizable) obstacles and solutions to include HIV testing recommendations in guidelines on HIV indicator diseases. This way, it may be possible not only to identify approaches to optimize HIV testing in different guidelines, but also to describe general ways to achieve a more interdisciplinary orientation of guidelines.

Overall, the HeLP study is expected to contribute an improved identification of HIV infections via optimized HIV testing service, and thus supports to achieve the 95-95-95 goals.

## Data Availability

Data sharing is not applicable to this article as no datasets were generated or analyzed at this stage of the research (study protocol).

## List of abbreviations

AIDS: Acquired immune deficiency syndrome
AOK: Allgemeine Ortskrankenkasse
AWMF: Association of the Scientific Medical Societies in Germany (“Arbeitsgemeinschaft der Wissenschaftlichen Medizinischen Fachgesellschaften”)
G-BA: German Federal Joint Committee (“Gemeinsamer Bundesausschuss“)
HeLP: HIV testing recommendations in guidelines and practice (“HIV-Testempfehlungen in Leitlinien und Praxis”)
HIV: Human immunodeficiency virus
RKI: Robert Koch Institute
SHI: Statutory health insurance(s)
WIdO: AOK Research Institute (“Wissenschaftliches Institut der AOK“)
